# Microstructure and Mechanical Properties of Fe-Rich Thixotropic Deep-Cavity Al-1.2Si-1.1Fe-0.8Zn Cylindrical Components with Inconsistent Wall Thickness

**DOI:** 10.3390/ma18040741

**Published:** 2025-02-07

**Authors:** Lingbo Kong, Jufu Jiang, Ying Wang, Xiaodong Zhang, Shanyong Tang, Tao Song

**Affiliations:** 1 National Key Laboratory for Precision Hot Processing of Metals, Harbin Institute of Technology, Harbin 150001, China; 24b909018@stu.hit.edu.cn (L.K.); 1190300724@stu.hit.edu.cn (X.Z.); 2 School of Materials Science and Engineering, Harbin Institute of Technology, Harbin 150001, China; 3 School of Mechatronics Engineering, Harbin Institute of Technology, Harbin 150001, China; 15012327683@163.com (S.T.); st1135268@163.com (T.S.)

**Keywords:** Fe-rich aluminum alloy, semi-solid processing, recrystallization behavior, mechanical properties

## Abstract

In this study, the thixotropic behavior of an Fe-rich Al-1.2Si-1.1Fe-0.8Zn aluminum alloy was thoroughly investigated. Firstly, ideal semi-solid billets were prepared through thermal deformation-induced isothermal spheroidization (TDIIS). The application of severe plastic deformation (SPD) via hot upsetting provided a strong driving force for recrystallization. As a result, the fibrous elongated grains gradually transformed into equiaxed grains following the TDIIS process. Overall, the grain size decreased with increasing deformation. However, as the temperature rose, the grain size initially decreased and then increased. The optimal conditions for the hot upsetting process were determined to be a temperature of 400 °C and a deformation of 50%. Under these conditions, the average grain size was 71.1 μm, and the shape factor was approximately 0.8, showcasing the excellent thixotropic properties of the semi-solid billets. Furthermore, the microstructure and mechanical properties of the thixotropic Al-1.2Si-1.1Fe-0.8Zn components were examined. These components, which were deep-cavity cylinders, exhibited an inconsistent wall thickness. Due to variations in the extrusion pressure, the grain morphology transitioned from dendritic at the upper part of cylinder wall to equiaxed at the bottom. This transition caused the elongation of the cylinder wall, resulting in it being lower than the cylinder bottom. During the thixoforming process, the equiaxed grains underwent deformation, and new recrystallized grains were formed. The multiscale synergy between the deformed grains, recrystallized grains and subgrains enabled the Fe-rich Al-1.2Si-1.1Fe-0.8Zn aluminum alloy to achieve well-balanced mechanical properties.

## 1. Introduction

Aluminum alloys have the advantages of low density, high specific strength, excellent corrosion resistance, and processing performance, and have gradually replaced steel and iron materials in the aerospace, automotive, and construction industries [1,2,3]. Typically, aluminum alloys can be processed through casting and forging methods [4,5]. Pursuing a trade-off between low deformation resistance and high mechanical properties remains a prominent research focus among scholars. Currently, semi-solid processing (SSP), which combines the benefits of casting and forging, has been extensively applied to aluminum alloy formation [6,7]. SSP operates within the semi-solid temperature range, where deformation resistance is remarkably low, and the semi-solid billets exhibit laminar flow. This process enables the metal to fill mold cavities effectively while minimizing the formation of defects, such as inclusions and porosity [8].

The technical pathway of SSP can be broadly divided into two stages: preparation of semi-solid billets and formation of semi-solids. The preparation of semi-solid billets with finely spherical or equiaxed grains is a prerequisite for SSP, as this microstructure endows the billets with excellent shear-thinning rheological properties [9]. Depending on the liquid fraction of the semi-solid billets, these preparation methods can be categorized into liquid-phase and solid-phase routes [10]. Slurries with a high liquid fraction exhibit good flowability and can be prepared through methods such as electromagnetic stirring [11], ultrasonic vibration [12], a serpentine channel pouring process [13], or a cooling slope [14]. However, these methods often face challenges, including container contamination and the inconvenience of the slurry transfer process, which collectively result in higher production costs.

Solid phase routes are employed to prepare semi-solid billets with a lower liquid-phase fraction. By regulating process parameters within the semi-solid temperature range, fine spherical or equiaxed grains can be obtained through recrystallization induced by thermal deformation, offering novel approaches to semi-solid billet preparation. Methods such as strain-induced melt activation (SIMA) [15], recrystallization, and remelting (RAP) [16], etc., have been demonstrated to be effective. The principle behind these methods lies in the storage of deformation energy after applying SPD, which provides the driving force for recrystallization [17]. Delbari Ragheb et al. [18] successfully eliminated defects, such as porosity and shrinkage, in as-cast 319 aluminum alloy prepared by SIMA. This approach resulted in a spherical semi-solid microstructure with enhanced mechanical properties.

Billets prepared by solid phase routes are typically processed through thixoforming. The optimum solid phase fraction (f_s_) for thixoforming is 0.5–0.7 [18]. When the f_s_ is lower than 0.5, the metal material becomes soft and unstable, making it difficult to clamp and handle. Conversely, when the f_s_ exceeds 0.7, the deformation resistance of the material increases significantly, reducing the lifespan of the mold [19]. Thixoforming generally consists of three steps [20]: (1) preparation of semi-solid billets with fine spherical or equiaxial grains, (2) reheating the billets, and (3) forming within the temperature range. Shabestari et al. [21] prepared semi-solid billets using SIMA and fabricated Al-10.5Si-3Cu-0.2Mg alloy components by thixoforming. These components exhibited excellent microstructures and mechanical properties compared to those obtained via casting. Liu et al. [22] designed a heterostructure to enhance the strength–ductility synergy of 2024Al via thixoforming. The improvement in mechanical properties was primarily attributed to hetero-deformation induced strengthening and hardening.

In this study, semi-solid billets were prepared using hot upsetting to accumulate recrystallization driving force and an isothermal treatment to induce recrystallization spheroidization, referred to as TDIIS. Deep-cavity cylindrical components with varying wall thickness were successfully produced using thixoforming, avoiding common defects such as shrinkage, voids, folding, and cracking by regulating the rheological behavior of the semi-solid billets. Subsequently, the variation in the microstructure and mechanical properties of the formed components was thoroughly analyzed. Finally, the optimal parameters for the billet preparation and forming processes were determined to guide production practices.

## 2. Materials and Methods

### 2.1. Raw Material

Aluminum bars obtained by hot extrusion were used in the present study. The chemical composition of the initial material is displayed in Table 1. Some studies have shown that Fe has very low solubility and diffusion rate in aluminum alloys [23]. High levels of Fe will tear the matrix and decrease fluidity, leading to brittle components and macro defects. In order to demonstrate the superior forming performance of SSP, the aluminum alloy with high Fe content was used in this work.

**Figure 1:**
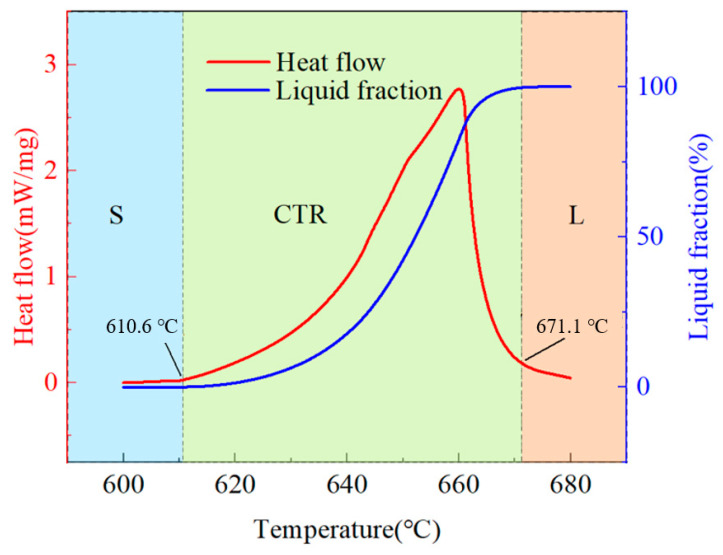
DSC curve of as-extruded aluminum alloy.

Figure 1 displays the DSC curve of initial material. The heating rate was 10 °C/min, with Ar flowing. The result showed that solid-phase temperature was 610.6 °C and the liquid phase temperature was 671.1 °C. The temperatures corresponding to high f_s_ were suitable for the preparation of semi-solid billets.

### 2.2. Preparation of Semi-Solid Billets

**Figure 2:**
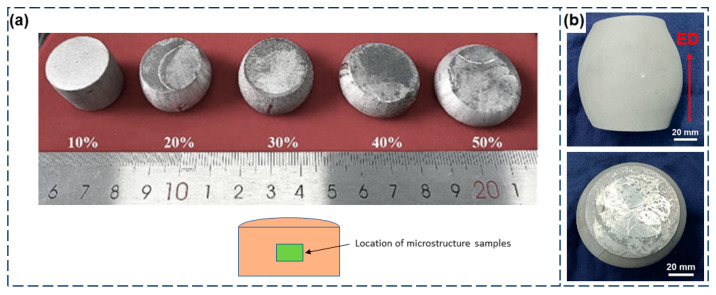
Semi-solid billets of Al-1.2Si-1.1Fe-0.8Zn aluminum alloy prepared by TDIIS: (**a**) billets with different upsetting deformations at 400 °C and (**b**) billets for thixoforming prepared by upsetting at 400 °C with 50% deformation.

Semi-solid billets were prepared using TDIIS. Hot upsetting was employed as a pre-treatment for the semi-solid billets to refine the raw material grains and store a significant amount of deformation energy, which provided the driving force for recrystallisation. The size of the original billets was Ø 20 mm × 20 mm. Before upsetting, graphite was sprayed on the upper and lower surfaces of billets to sufficiently reduce friction. The parameters for hot upsetting are shown in Table 2. Figure 2 shows the appearance of the hot upsetting samples after different deformations, with no cracks or other defects observed on the surface.

### 2.3. Thixoforming Experiment

**Figure 3:**
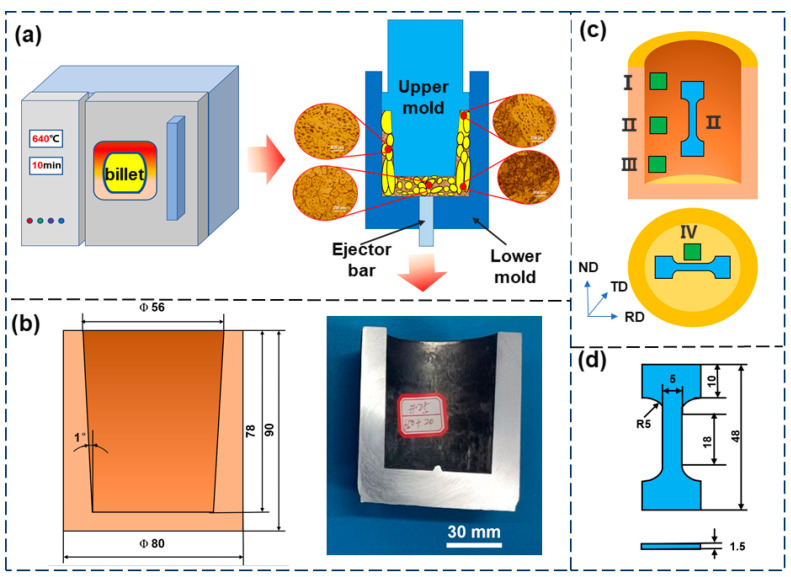
The schematic of the deep-cavity cylindrical part (**b**) produced by the thixotropic forming process (**a**), along with the sampling positions (**c**) and dimensions of the tensile specimen (**d**).

The holding process played a crucial role in the semi-solid thixoforming experiments. The parameters of the thixoforming experiment, as determined by the DSC curve, are shown in Table 3. Figure 3a shows the schematic of the main process for the thixoforming experiment. The semi-solid billets were placed in the holding furnace for 10 min at 640 °C, and then immediately transferred to the 200-ton hydraulic press for thixoforming. No holes, cracks, or other defects were observed in the deep-cavity cylindrical components with inconsistent wall thicknesses, indicating excellent forming quality (Figure 3b).

### 2.4. Characterization of Microstructure and Mechanical Properties

Due to the differences in pressure and friction between the cylinder wall and the cylinder bottom, separate samples were taken for analysis. Figure 3c illustrates the sampling scheme. The specimens were sequentially sanded using sandpapers of grit sizes 400#, 800#, 1200#, 1500#, and 2000#, followed by mechanical polishing prior to microstructure observation. Keller’s reagent (volume fraction 2.5% HNO_3_ + 1.5% HCl + 1% HF + 95% H_2_O) was used for etching. Metallographic microscopy was employed to analyze the microstructure morphology. Scanning electron microscopy (SEM) and energy dispersive X-ray spectroscopy (EDS) were used to analyze the second-phase morphology and specific chemical composition. Dynamic recrystallization occurred during thixoforming and electron backscatter diffraction (EBSD) analysis was necessary. After mechanical polishing, the samples for EBSD analysis required electrolytic polishing using a mixture of 20% HClO_4_ and 80% alcohol to remove surface stresses. Tensile tests were carried out with a 20 kN universal tensile testing machine with the tensile specimen dimensions provided in Figure 3d. The tests were conducted at least three times to ensure the repeatability of the experimental results.

## 3. Results and Discussion

### 3.1. Microstructural Evolution of Semi-Solid Billets Prepared by TDIIS

**Figure 4:**
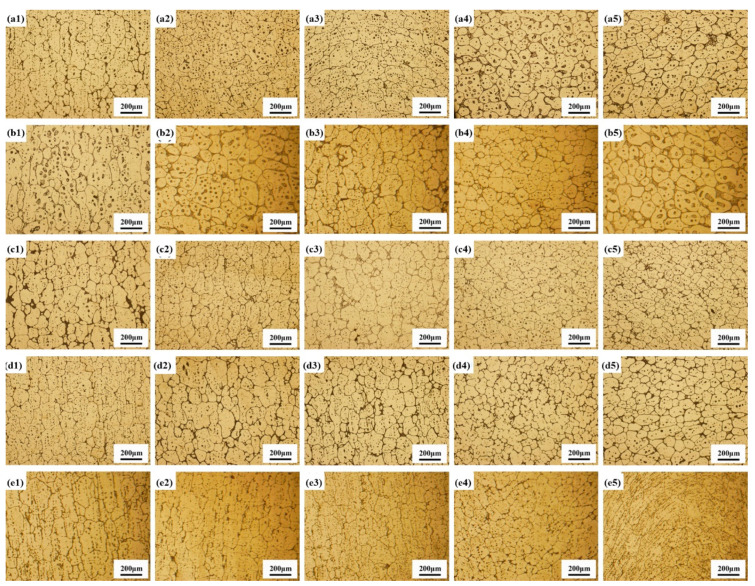
Microstructure of semi-solid billets prepared by TDIIS with deformation of 10% (**a1**–**e1**), 20% (**a2**–**e2**), 30% (**a3**–**e3**), 40% (**a4**–**e4**), and 50% (**a5**–**e5**) at 380 °C (**a1**–**a5**), 390 °C (**b1**–**b5**), 400 °C (**c1**–**c5**), 410 °C (**d1**–**d5**), and 420 °C (**e1**–**e5**), respectively.

Figure 4 shows the microstructure of the semi-solid billets prepared by TDIIS at different deformations and holding temperatures. The fine rounded equiaxed grains with shear-thinning rheological properties were the ideal microstructure for SSP [24]. SPD provided the driving force required for recrystallization. The more intense the plastic deformation, the greater the accumulation of energy required for recrystallization nucleation and grain boundary expansion, which led to a higher frequency of recrystallization occurrences. With only 10% deformation, elongated and equiaxed grains were mixed. The grain size was uneven, and fine equiaxed grains were formed mainly at grain boundaries (Figure 4(a1–e1)). With the increase in deformation, the uniformity of the microstructure was significantly improved. On one hand, SPD stored a large amount of energy, which provided enough driving force for recrystallization. On the other hand, SPD accumulated a lot of dislocations, which promoted the nucleation of subgrains [25].

**Figure 5:**
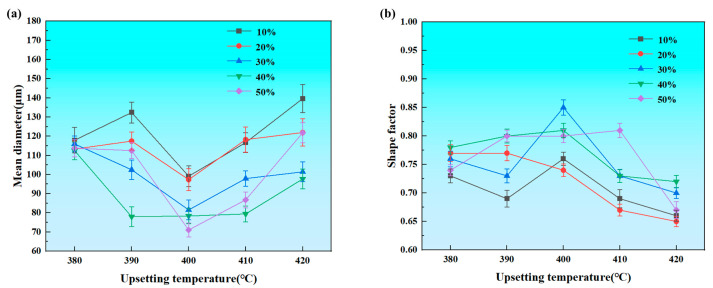
Variation in average grain size (**a**) and shape factor (**b**) of aluminum alloys under different upsetting temperatures and deformation.


In order to visualize the trend in grain size variation, the statistical results for the grain size and shape factor of each specimen in Figure 4 are shown using Image Pro Plus (IPP) software in Figure 5. The average grain size was determined by Equation (1), and the shape factor was determined by Equation (2) [26].

(1) $$D=\frac{\sum_{i=1}^{N}\sqrt{\frac{4A}{\pi }}}{N}$$

Here, D is average grain diameter (μm), N is number of grains counted, and A is grain area (μm^2^).

(2) $$F=\frac{\sum_{i=1}^{N}\frac{4\pi A}{P^{2}}}{N}$$

Here, F is shape factor, N is number of grains counted, P is grain perimeter (μm), and A is grain area (μm^2^).

Over all, the grain size first decreased and then increased when the temperature increased. Raising the temperature was beneficial for the nucleation and growth of the recrystallized grains [27]. This was because as the temperature increased, dislocations, subgrain boundaries, and grain boundary motion became easier, which had a profound impact on the recrystallization process [28]. However, excessively high temperatures could lead to grain coarsening. The growth of the grains resulted in an increase in the average grain size. The shape factor reflected the degree to which the particle shape deviated from a spherical shape. The higher the shape factor, the more pronounced the spheroidization effect. Fine-rounded, semi-solid spherical grains were confirmed to have a shear-thinning rheology [29]. Li et al. pointed out that a higher deformation energy leads to a lower recrystallization temperature and finer recrystallized grains [30]. At 400 °C, as the deformation increased, the recrystallized grains became finer. The grains size reached a low level, with a minimum of 71.1 μm at 50% deformation. Meanwhile, the shape factor was above 0.80, indicating good spheronization. Therefore, the upsetting temperature required to prepare semi-solid billets was determined to be 400 °C, and the deformation was determined to be 50%.

### 3.2. Microstructural Evolution of Deep-Cavity Al-1.2Si-1.1Fe-0.8Zn Cylindrical Components

**Figure 6:**
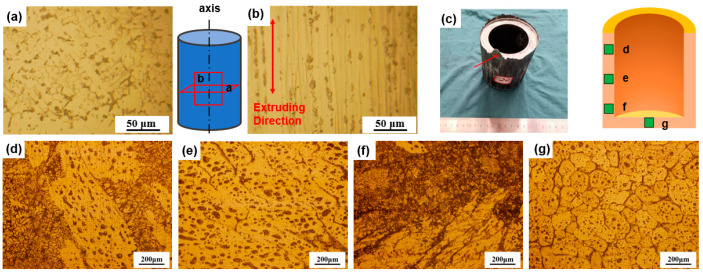
Comparison of microstructure of aluminum alloy bars and thixotropic-formed cylindrical parts (holding at 645 °C for 20 min) in different directions of extrusion: microstructure (**a**) perpendicular to ED and (**b**) along ED; (**d**–**g**) microstructure observed at positions indicated in (**c**).

Figure 6 shows a comparison of the microstructures before and after thixoforming. The grains of the original extruded bar were elongated in the extrusion direction (ED), with no visible grain boundaries. In the direction perpendicular to the ED, a small number of dendritic grains were observed, which were refined and compacted, resulting in an overall dense microstructure. On the upper side of the cylinder wall, elongated and eutectic microstructures coexisted (Figure 6d). The grains and the liquid phase in the middle of the cylinder wall were elongated along the cylinder’s axis, with some grain boundaries merging (Figure 6e). At the bottom of the cylinder wall, no intact grains were observed, and the grain boundaries had completely disappeared (Figure 6f). This change in grain boundaries indicated that the liquid phase fraction decreased from the top to the bottom of the cylinder wall. In contrast, the microstructure at the bottom of the cylinder showed a morphology with fine and uniform equiaxed grains (Figure 6g).

The coexistence of solid and precipitated liquid phases is a typical process in SSP [31]. A related theory states that a new interface will form when the new phase is precipitated in the original phase [32]. By analyzing the microstructures of the formed part and the extruded bar, it was reasonable to identify the second phase within the grains, and the liquid phase at the grain boundaries of the newly precipitated phases. The formation of the new phase required a sufficient free-energy difference from the original phase. This difference could be achieved, on one hand, by increasing the degree of undercooling, and on the other hand, by applying pressure to provide the nucleation driving force. The upper end of the cylinder wall was in contact with the upper end of the punch-pin, resulting in faster heat dissipation. This caused the degree of undercooling in this part to be greater than in other areas, thereby preferentially meeting the nucleation conditions. Therefore, a large number of newly dendritic grains were generated in this part (Figure 6d).

**Figure 7:**
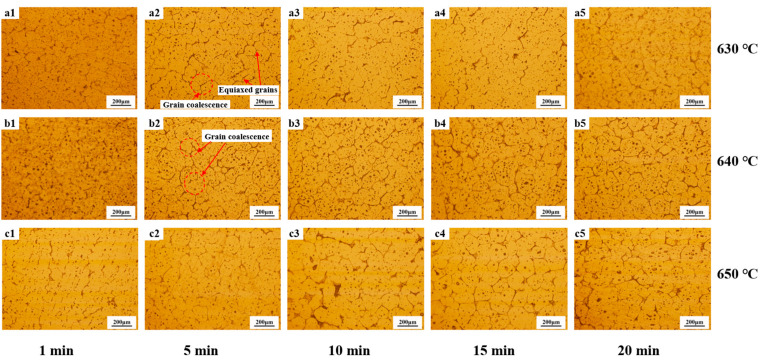
The microstructure of the cylinder bottom with a holding temperature of 630 °C (**a1**–**a5**), 640 °C (**b1**–**b5**), and 650 °C (**c1**–**c5**), and a holding time of 1 min (**a1**–**c1**), 5 min (**a2**–**c2**), 10 min (**a3**–**c3**), 15 min (**a4**–**c4**), and 20 min (**a5**–**c5**).

**Figure 8:**
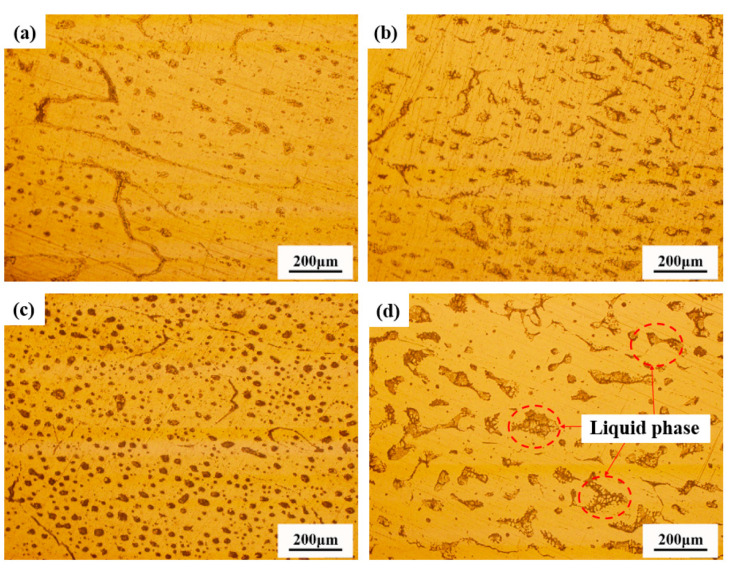
Microstructure of cylinder wall after 20 min of holding at temperatures of 630 °C (**a**), 635 °C (**b**), 640 °C (**c**), and 650 °C (**d**).

Figure 7 illustrates the variation in the grain morphology at the cylinder bottom with varying temperatures and holding times. Initially, the microstructure predominantly consisted of equiaxed grains. As the holding time increased, the degree of grain spheroidization progressively enhanced. However, grain coarsening became evident at 650 °C for a 20 min holding time. Figure 8 depicts the microstructural changes in the middle section of the cylinder wall after holding for 20 min at different temperatures. With increasing holding temperatures, the liquid phase precipitation at the grain boundaries and within the grains became more pronounced. The microstructure was primarily composed of dendritic grains.

**Figure 9:**
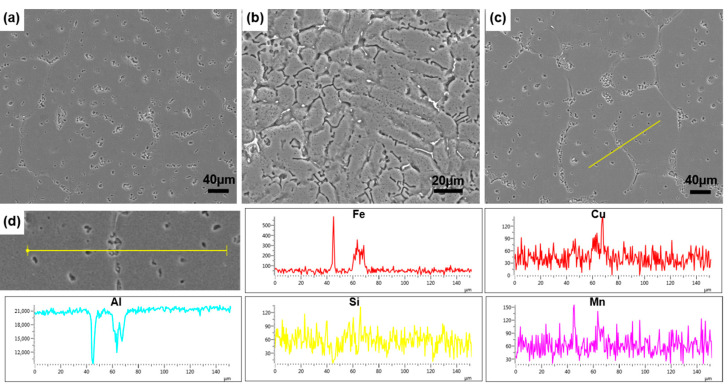
SEM results at bottom of cylinder after holding at (**a**) 640 °C for 5 min and (**c**) 640 °C for 20 min, and at the cylinder wall (**b**) after holding at 645 °C for 20 min; EDS results at bottom of cylinder after holding at (**d**) 640 °C for 20 min.

In order to reveal the element distribution of the second phase, SEM and EDS tests were conducted, and the results are shown in Figure 9. The cylinder wall exhibited a morphology with numerous dendrites, while the bottom of the cylinder consisted of equiaxed grains, which was consistent with the metallographic results. Second-phase particles were distributed in a dot-like pattern at the grain boundaries and within the grains, and primarily contained Fe. The distribution of the Mn elements was consistent with the Fe elements, which could alleviate the tearing effect [33]. In this study, Fe was added in excess to reflect the good filling performance of semi-solid thixoforming. Fe elements typically exhibit a dendritic distribution [34]; however, the original hot-extruded bars underwent SPD again through TDIIS. Additionally, pressure was also applied to the semi-solid billets during the thixoforming. As a result, these deformations refined the Fe-rich second phase. Huang et al.’s research indicates that a refined Fe phase achieved by SPD can improve the material’s microstructure and enhance its mechanical properties [23]. These refined Fe particles could also act as pinning sites for dislocations, thereby refining the grains.

### 3.3. Mechanical Properties of Deep-Cavity Al-1.2Si-1.1Fe-0.8Zn Cylindrical Components

**Figure 10:**
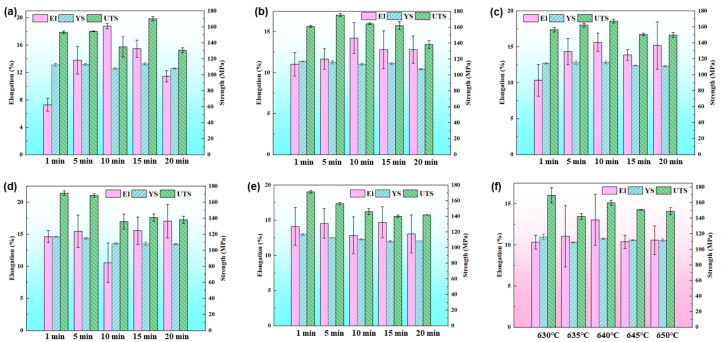
Variations in mechanical properties of cylinder bottom and wall: (**a**–**e**) mechanical properties of cylinder bottom formed at (**a**) 630 °C; (**b**) 635 °C; (**c**) 640 °C; (**d**) 645 °C; and (**e**) 650 °C; (**f**) mechanical properties of cylinder wall formed at 20 min with different temperatures.

Samples were taken from the cylinder wall and the bottom of the components for testing of their tensile mechanical properties, and the experimental results are shown in Figure 10. The mechanical properties were influenced by three main factors. First, the differences in forming temperatures led to an inconsistent liquid phase fraction, and the liquid phases polarized at the grain boundaries were shown to be detrimental to the mechanical properties [35]. Secondly, the varying pressures at different locations led to inconsistent grain deformation, with significant work hardening in the areas of larger deformation. Third, the different holding times led to inconsistent grain sizes. Fine grains obtained with a reasonable holding time could effectively improve the mechanical properties [36]. At 630 °C and 635 °C, the elongation showed a trend of first increasing and then decreasing with an extension of the holding time for the cylinder bottom. Considering elongation (El), yield strength (YS), and ultimate tensile strength (UTS) comprehensively, the mechanical properties were optimal with 15 min of holding. With the increase in temperature, the mechanical properties of the cylinder bottom reached their best performance with 10 min of holding at 640 °C, 5 min of holding at 645 °C, and 1 min of holding at 650 °C, separately. As the temperature increased, more of the liquid phase precipitated. Atkinson et al. have confirmed that liquid phase precipitation is particularly severe at the grain boundaries, so the increase in the liquid phase has a more significant impact on the mechanical properties [37].

Figure 10f shows the variation of the tensile mechanical properties at the cylinder wall when held for 20 min at different temperatures. At 630 °C, the strength was higher due to the greater deformation resistance and significant work hardening effect. As the microstructure became uniform, the El reached its maximum at 640 °C, but the strength slightly decreased due to liquid phase precipitation (Figure 8c). As the temperature further increased, the amount of the liquid phase increased, and the grains became coarser, leading to a decrease in their mechanical properties (Figure 6e and Figure 8d).

**Figure 11:**
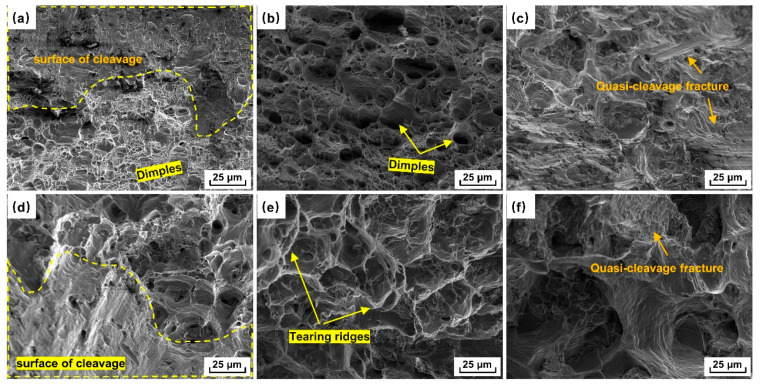
Fracture morphology of some tensile specimens: sampling at cylinder bottom formed at (**a**) 640 °C for 1 min, (**b**) 640 °C for 10 min, and (**c**) 640 °C for 20 min and at cylinder wall formed at (**d**) 630 °C for 20 min, (**e**) 640 °C for 20 min, and (**f**) 650 °C for 20 min, respectively.

Figure 11a–c display the fracture characteristics observed by SEM at the cylinder bottom after holding at 640 °C for 1 min, 10 min, and 20 min, respectively. After holding for 1 min, the fracture surface mainly consisted of dimples and surface of cleavage, showing a ductile–brittle mixed fracture (Figure 11a). After holding for 10 min, the fracture surface exhibits numerous dimples, indicating ductile fractures (Figure 11b). After holding for 20 min, a small number of quasi-cleavages appeared, and the strength slightly decreased (Figure 11c) [38]. Studies have shown that the coarse Fe-rich phase can act as the source of crack initiation, adversely affecting the mechanical properties [39]. Thanks to the grain refinement effect of SPD, the fine Fe-rich phase morphology in this study effectively mitigated its inherent tearing effect.

Figure 11d–f shows the tensile fracture morphology of some specimens from the cylinder wall after holding at 630 °C, 640 °C, and 650 °C for 20 min. The tearing ridges showed a ductile fracture for the tensile specimen formed when holding at 640 °C for 20 min (Figure 11e), which was consistent with the results for the tensile mechanical properties (Figure 10f). However, some brittle fracture characteristics, such as the surface of cleavage, were observed at 630 °C and 650 °C (Figure 11d,f).

### 3.4. Recrystallization of Deep-Cavity Al-1.2Si-1.1Fe-0.8Zn Cylindrical Components

**Figure 12:**
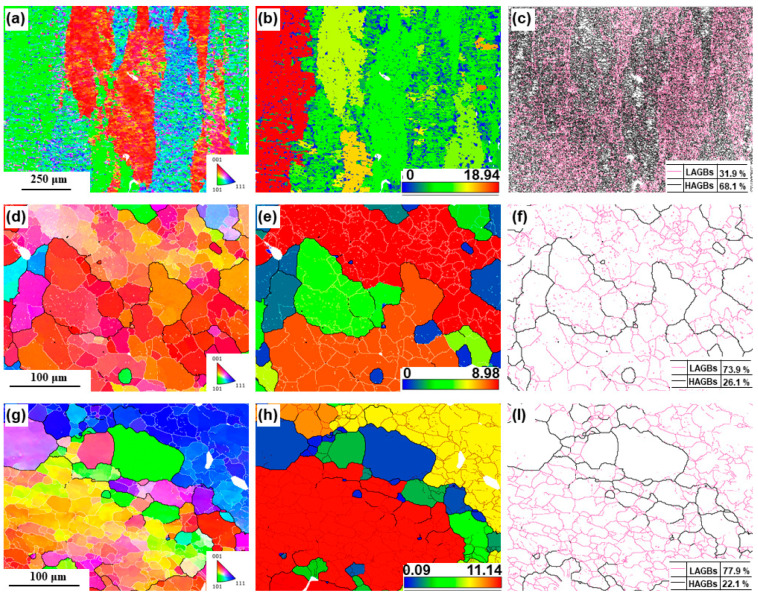
EBSD results of aluminum bar after hot upsetting, and component under the forming condition of 640 °C for 10 min: (**a**) IPF map, (**b**) GOS map, and (**c**) grain boundary map of aluminum bar after hot upsetting; (**d**) IPF map, (**e**) GOS map, and (**f**) grain boundary map at the position of cylinder wall; (**g**) IPF map, (**h**) GOS map, and (**l**) grain boundary map at the position of cylinder bottom. Among them, colors in IPF indicate crystal directions parallel to normal direction of the sample.

Figure 12 shows the EBSD results for the aluminum bar after hot upsetting and the cylinder wall and bottom of component after holding at 640 °C for 10 min. The inverse pole figure (IPF) map reflects the orientation relationship of the grains. The grain-based grain orientation spread (GOS) reflects the distribution of deformed grains (GOS values larger than 3°), subgrains (GOS values of 1.8–3°), and recrystallized grains (GOS values of 0–1.8°) [40,41]. The grain boundary map reflects the distribution of high angle grain boundaries (HAGBs, misorientations larger than 15°) and low angle grain boundaries (LAGBs, misorientations smaller than 15°) [42]. For the Fe-rich aluminum alloy bar after hot upsetting, the grains were mainly composed of elongated and deformed grains (Figure 12a,b). Some fine recrystallized grains were distributed at the grain boundaries of the deformed grains. There were many LAGBs inside the deformed grains, and even some HAGBs were distributed among them (Figure 12c). This indicates that SPD accumulated a significant amount of energy. Interestingly, the subgrains of the components exhibited significant orientation differences despite being within the same grain, which resulted from the rotation of the dislocations between adjacent subgrains (Figure 12d,g). Studies have shown that the orientations of recrystallized grains became random [43]. As one of the sources of recrystallized grains, the orientation differences among the subgrains reflect, to some extent, the progress of recrystallization. The cylinder wall and bottom were composed of deformed grains, with a large number of fine equiaxed subgrains present inside the deformed grains. A small number of fine dynamic recrystallized grains were generated at the grain boundaries (Figure 12e,h). In addition, some of the subgrains inside the grains transformed into equiaxed grains. These two grain formation mechanisms differed from each other. The recrystallized grains at the grain boundaries were formed by the bending of the originally elongated deformed grains, mainly due to the difference in dislocations within the two deformed grains. The recrystallized grains inside the grains were formed as the dislocations were pinned by second-phase particles and other obstacles. Insoluble Fe plays an active role in pinning dislocations and refining grains. At the cylinder wall and bottom, the content of LAGBs was relatively significant (Figure 12f,l). During the SSP, dynamic recrystallization was highly effective, primarily occurring through subgrain transformation. The abundant LAGBs within the deformed grains significantly contributed to enhancing the material’s plasticity.

**Figure 13:**
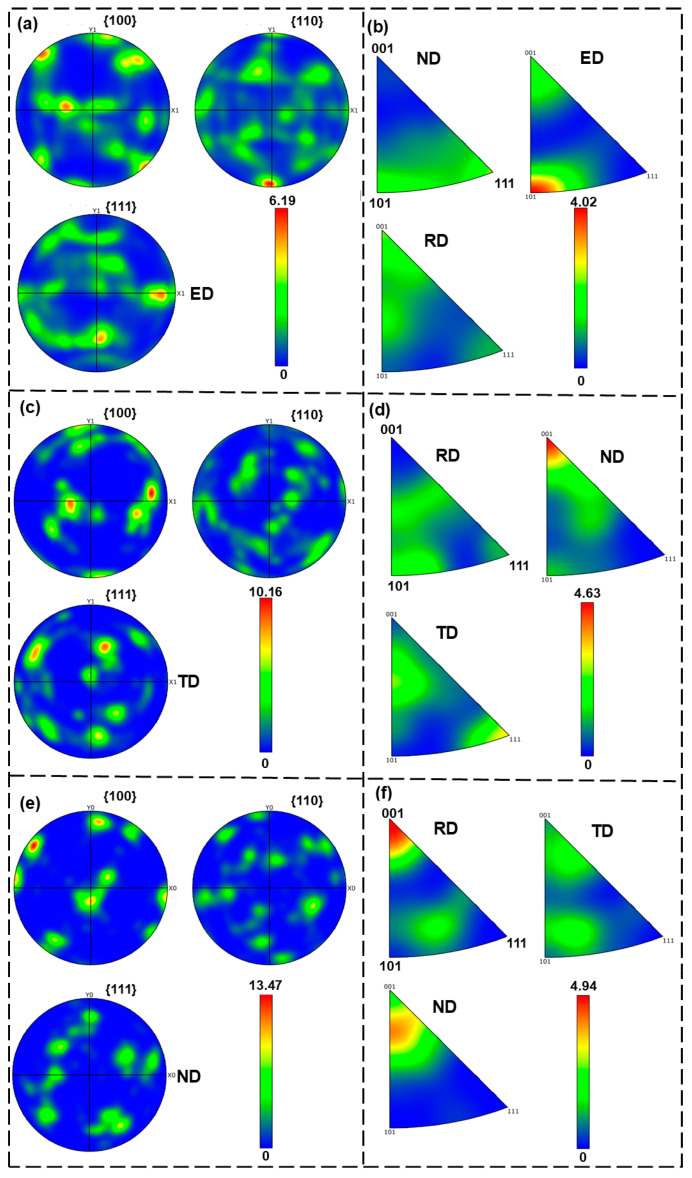
PF and IPF of aluminum bar after hot upsetting, and component under forming condition of 640 °C for 10 min: (**a**) PF and (**b**) IPF of aluminum bar after hot upsetting; (**c**) PF and (**d**) IPF at position of cylinder wall; (**e**) PF and (**f**) IPF at position of cylinder bottom.

Figure 13 illustrates the pole figures (PFs) and IPFs of the aluminum bar after hot upsetting, and the component under the forming condition of 640 °C for 10 min. For aluminum alloy bars after hot upsetting, the preferred orientation of grains in the ED direction was more pronounced. The maximum PF intensities at the cylindrical wall and bottom were 10.16 and 13.47, respectively, indicating a pronounced texture after thixoforming. This suggests that the grain orientation at the bottom is more concentrated than at the cylindrical wall. The IPF further confirmed the significant texture characteristics of the material. Generally speaking, according to the Hall–Petch equation, the smaller the grain size, the higher the strength [44]. However, in this study, not all recrystallization led to strengthening. Due to the experimental temperature being within the semi-solid range, the wetting effect of the liquid phase weakened the strength of the grain boundaries, so the recrystallization generated at the grain boundaries did not result in strengthening. Inside the deformed grains, the recrystallized grain boundaries were formed by dislocation entanglement and rotation, and these grain boundaries were in a strengthened state. The research results indicate that the number of subgrains inside the grains was much larger than the number of recrystallized grains at the grain boundaries. At the same time, the relatively concentrated grain orientation indicated a good work-hardening effect. Therefore, overall, recrystallization played a strengthening role in the material.

**Figure 14:**
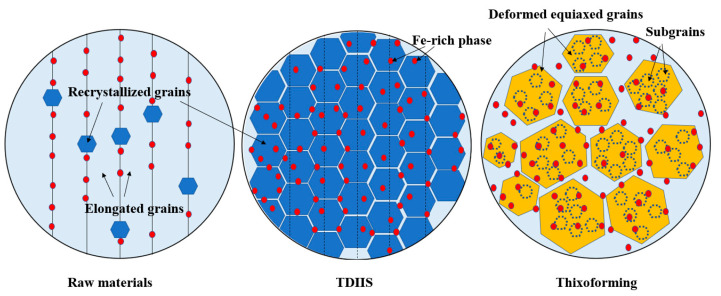
Schematic diagram of recrystallization in TDIIS and thixoforming.

Figure 14 summarizes the recrystallization behavior from semi-solid billet preparation to thixoforming. The recrystallization behavior of the semi-solid billets was the most pronounced, primarily because a large number of dislocations had accumulated during the initial extrusion process. SPD was applied again through TDIIS, which caused a large amount of deformation energy to accumulate within the material, leading to the formation of fine equiaxed recrystallized grains at a semi-solid temperature. Recrystallization behavior was not obvious during the thixoforming. The recrystallization driving force that accumulated from the SPD was consumed during the semi-solid billet preparation, resulting in a significant reduction in the content of LAGBs in the billet. During the thixoforming process, the recrystallization driving force mainly came from the extrusion pressure applied by the mold. Recrystallization mainly occurred through two mechanisms: grain boundary bowing and subgrain transformation within the grains. The recrystallization formed during billet preparation had already transformed into deformed grains under the action of pressure. Some researchers have pointed out that the mechanical properties of the billet are significantly reduced due to the accumulation of the liquid phase at the grain boundaries [15]. This spherical semi-solid microstructure, encapsulated by the liquid phase, greatly improved the material’s flow and filling performance. However, there were a large number of subgrain boundaries within the deformed grains, and the coordination between the deformed grains and subgrains enhanced the material’s mechanical properties.

In summary, we designed an Fe-rich aluminum alloy and conducted research on the preparation of semi-solid billets and thixoforming. The optimal preparation process for semi-solid billets was determined to be a 50% deformation at 400 °C using TDIIS. Under this condition, the grain size of the billet was 71.1 μm, and the shape factor exceeded 0.8. Subsequently, thixoforming experiments for the Fe-rich deep-cavity Al-1.2Si-1.1Fe-0.8Zn cylindrical components were conducted. We systematically analyzed the microstructure and mechanical properties of the cylinder wall and bottom. The results showed that the grains at the cylinder bottom were predominantly equiaxed grains, while those at the cylinder wall were mainly dendritic grains. As the holding time increased, the grains gradually coarsened. Fe was found to exhibit a dotted distribution in the aluminum matrix and at grain boundaries, with Mn showing a similar distribution pattern, effectively mitigating the detrimental effect of Fe on the Al matrix. The mechanical properties results indicated that the El of the cylinder bottom initially increased and then decreased with the extension of the holding time at temperatures of 630 °C and 635 °C, achieving optimal mechanical properties with 15 min of holding. As the temperature increased, the optimal mechanical properties of the cylinder bottom were observed at 10 min of holding at 640 °C, 5 min at 645 °C, and 1 min at 650 °C. The El of the cylinder wall reached its maximum at 640 °C, but the strength slightly decreased due to liquid phase precipitation. Finally, EBSD technology was used to reveal the changes in recrystallization behavior. The results showed that after holding at 640 °C for 10 min, a large number of subgrain boundaries were present within the grains at both the cylinder wall and bottom. The content of LAGBs was relatively high in these regions. The maximum pole figure intensities at the cylinder wall and bottom were 10.16 and 13.47, respectively, indicating that the grain orientation at the cylinder bottom was more concentrated than at the cylinder wall.

## 4. Conclusions

Fe-rich components are typically difficult to form due to the detrimental effects of Fe on the matrix. This study utilized the TDIIS method to prepare semi-solid billets, combined with thixoforming technology, which effectively resolved the thermal cracking issues in Fe-rich components. During this process, the microstructural characteristics of the semi-solid billets were thoroughly investigated, and the optimal billet preparation parameters were determined. By controlling the forming temperature and time, the microstructure and mechanical properties of the formed components were further optimized. Finally, the recrystallization mechanism during the forming process was summarized, leading to the following main conclusions:

1. Ideal semi-solid billets were successfully prepared through TDIIS. The variations in microstructural morphology and shape factor under different temperature and deformation amounts were systematically studied. SPD provided sufficient driving force for the recrystallization process. With an increase in the deformation amount, the uniformity of the microstructure improved significantly. At 400 °C, as the deformation amount increased, the recrystallized grains became progressively finer, reaching a minimum grain size of 71.1 μm at 50% deformation. Meanwhile, the shape factor exceeded 0.80, indicating excellent spheroidization. Therefore, the optimized preparation conditions were determined to be a preparation temperature of 400 °C and a deformation amount of 50%.
2. Deep-cavity Al-1.2Si-1.1Fe-0.8Zn cylindrical components were prepared under different process parameters through thixoforming experiments. The microstructural analysis revealed a gradual transition in the microstructure from dendrites to spheroidal grains from the cylinder wall to the bottom. Simultaneously, the amount of precipitated liquid phase gradually decreased. Second-phase particles, primarily rich in Fe, were observed to be distributed in a point-like pattern along grain boundaries and within grains. The distribution of Mn was consistent with that of Fe, which helped alleviate tearing effects in the material. As the forming temperature increased, the amount of precipitated liquid phase gradually increased under the same holding time.
3. Tensile mechanical property tests were conducted on the cylinder wall and bottom of selected components. Under conditions of 630 °C and 635 °C, the El of the cylinder bottom exhibited a trend of initially increasing and then decreasing with the extension of the holding time, with optimal mechanical properties achieved at 15 min of holding. As the temperature increased, the mechanical properties of the cylinder bottom reached their optimal state at 10 min of holding at 640 °C, 5 min at 645 °C, and 1 min at 650 °C. At the cylinder wall, the El reached its maximum at 640 °C, but the strength was slightly decreased due to liquid phase precipitation.
4. The EBSD results indicated that a large number of subgrain boundaries existed within the grains at the cylindrical wall and the bottom of the components after holding at 640 °C for 10 min. The subgrains exhibited significant orientation differences due to the rotation of the dislocations between adjacent subgrains, serving as one of the primary sources of recrystallization. At the cylindrical wall and bottom, the content of LAGBs was relatively high. The maximum PF intensities at the cylindrical wall and bottom were 10.16 and 13.47, respectively, indicating that the grain orientation at the bottom was more concentrated than at the cylindrical wall.

## Figures and Tables

**Table 1:** Chemical composition of as-extruded aluminum alloy.

**Element**	Si	Mg	Zn	Fe	Cu	Mn	Cr	Al
**wt** %	1.237	0.703	0.824	1.064	0.504	0.346	0.144	balance

**Table 2:** Parameters of hot upsetting for Al-1.2Si-1.1Fe-0.8Zn aluminum alloy.

Serial Number	Holding Temperature (°C)	Deformation (%)	Serial Number	Holding Temperature (°C)	Deformation (%)
1	380	10	14	400	40
2	380	20	15	400	50
3	380	30	16	410	10
4	380	40	17	410	20
5	380	50	18	410	30
6	390	10	19	410	40
7	390	20	20	410	50
8	390	30	21	420	10
9	390	40	22	420	20
10	390	50	23	420	30
11	400	10	24	420	40
12	400	20	25	420	50
13	400	30			

**Table 3:** Parameters for isothermal treatment of Al-1.2Si-1.1Fe-0.8Zn aluminum alloy.

Serial Number	Holding Temperature (°C)	Holding Time (min)	Serial Number	Holding Temperature (°C)	Holding Time (min)
1	630	1	14	640	15
2	630	5	15	640	20
3	630	10	16	645	1
4	630	15	17	645	5
5	630	20	18	645	10
6	635	1	19	645	15
7	635	5	20	645	20
8	635	10	21	650	1
9	635	15	22	650	5
10	635	20	23	650	10
11	640	1	24	650	15
12	640	5	25	650	20
13	640	10			

## Data Availability

The original contributions presented in this study are included in the article, and further inquiries can be directed to the corresponding author.
